# Physical Activity among Spanish Undergraduate Students: A Descriptive Correlational Study

**DOI:** 10.3390/ijerph16152770

**Published:** 2019-08-02

**Authors:** Jorge Acebes-Sánchez, Ignacio Diez-Vega, Gabriel Rodriguez-Romo

**Affiliations:** 1 Faculty of Health Sciences, Universidad Francisco de Vitoria (UFV), Pozuelo de Alarcón, 28223 Madrid, Spain; 2 Faculty of Physical Activity and Sport Sciences (INEF), Universidad Politécnica de Madrid (UPM), 28040 Madrid, Spain; 3 Faculty of Sport Sciences, Universidad Europea de Madrid (UEM), Villaviciosa de Odón, 28670 Madrid, Spain; 4 Centro de Investigación Biomédica en Red Fragilidad y Envejecimiento Saludable (CIBERFES), 28029 Madrid, Spain

**Keywords:** physical activity, leisure-time physical activity, GPAQ, undergraduate students, recommendations

## Abstract

Achieving the recommended levels of physical activity (PA) is associated with better health. Despite this, most undergraduate students report low levels of PA. This study aimed to assess the achievement of recommended PA levels in a wide sample of undergraduate students from Madrid (*N* = 2960). Overall PA and leisure-time PA (LTPA) were measured with the Global Physical Activity Questionnaire (GPAQ). Descriptive analyses and logistic regression were performed. It was revealed that 22.4% and 55.6% of overall PA and LTPA, respectively, did not achieve World Health Organization (WHO) recommendations. When PA was measured as overall PA, the achievement of the recommended level was positively predicted by male sex; a Body Mass Index (BMI) indicative of normal weight or being overweight; spending less time sitting or reclining; work; and studying health science, social sciences, engineering, or architecture (all *p* < 0.001; *r*^2^ = 0.075). Using LTPA, the positive predictors of achieving recommended PA levels were male sex, having a BMI indicative of normal weight or being overweight, work, studying at a public university, and studying health science (*r*^2^ = 0.048). These findings suggest that universities should implement strategies to promote PA.

## 1. Introduction

According to the “Special Eurobarometer 472: Sport and Physical Activity” data recently published by the European Commission [1], 46% of the European population never participates in exercise or does sports. This figure has not improved since 2009. It is widely recognized that physical inactivity is one of the risk factors for noncommunicable diseases such as coronary heart disease, type 2 diabetes, metabolic syndrome, and some types of cancer [2]. Similarly, adequate physical activity (PA) levels are associated with better mental health and social wellness [3,4,5,6]. Physical inactivity is currently a global public health problem, as it is the fourth biggest risk factor for global mortality [7] and is responsible for 1.6 million deaths per annum [8]. Lee, Shiroma, Lobelo, Puska, Blair, and Katzmarzyk [2] have suggested that 5.3 million deaths could be prevented if physically inactive individuals achieved recommended PA levels. The World Health Organization (WHO) recommends that adults aged 18–64 years should do at least 150 min of moderate-intensity aerobic physical activity or at least 75 min of vigorous-intensity aerobic physical activity per week or an equivalent combination of moderate- and vigorous-intensity activity [7]. According to the WHO, PA can be measured in terms of time spent, intensity, and the metabolic equivalent of task (MET). Three domains of PA are recognized: leisure-time PA (LTPA), occupational PA (OPA; PA undertaken in the course of a paid or unpaid job, studies, housework, or a job search), and commuting PA (CPA; walking or cycling to work). The result of the addition of the three domains is the overall PA [7].

Undergraduate students are in a transitional phase of life that entails many changes in their daily habits. They have moved from a structured environment (school) to a relatively unstructured environment (university) [9]. This transition reduces overall PA levels [10,11,12,13].

A study of undergraduate students from 23 different countries [14] reported that 41.4% were not achieving the recommended levels of PA, based on an assessment of overall PA. Cocca et al. [11] found that the proportion of inactive Spanish young adults was 60%. Another study [15] found this proportion higher (72.6%). Furthermore, sociodemographic variables have been related to overall PA and LTPA [14,16,17,18]. Thus, younger undergraduate students or being a man were related to better PA levels. Academic variables have also been related to PA [19,20]. For instance, there was a positive association between academic achievement and PA. On the other hand, other studies have found a negative association between sedentary behavior and PA [21,22].

It seems, therefore, that promoting PA among the student population specifically, as well as the general population, should be a public health priority. Promoting LTPA might be a good way to increase overall PA levels, as this is the domain in which individuals have the most control over their activity. However, the design of specific strategies for PA promotion in this population group requires accurate information about PA levels and their possible relationships with different variables. Although several research papers have already been developed for this purpose, we are not aware of any recent study that has provided differentiated information on the levels of overall PA and LTPA in a large sample of university students from different Spanish universities.

Based on the importance of PA in undergraduate students, its association with health, and the low levels of PA in this population [1,15], this study had three aims: (1) to describe PA levels in male and female undergraduate students from Madrid; (2) to assess the proportion of students achieving recommended PA levels (based on overall PA and LTPA); and (3) to analyze the relationships between the achievement of recommended PA levels and sociodemographic, academic, and lifestyle variables.

## 2. Materials and Methods

### 2.1. Design and Participants

We conducted a descriptive and cross-sectional study to analyze overall PA and LTPA. The effects of sociodemographic, academic, and lifestyle variables on PA and LTPA were also studied.

The participants were undergraduate students from public and private universities in Madrid: 2960 undergraduate students completed the online questionnaire designed for the investigation. The sample was disproportionate: A stratified sample design based on the type of university (public or private) and academic discipline of the student (social and juridical sciences, engineering and architecture, arts and humanities, health sciences, physical and life sciences) was carried out. Participation was voluntary and confidential, and informed consent was obtained from participants before they completed the survey.

Ethical approval for this study was obtained from the Ethics Research Committee of the Universidad Francisco de Vitoria (40/2018). The study was performed in accordance with the Declaration of Helsinki.

### 2.2. Data Collection

Lecturers from all universities (6 public and 7 private) in Madrid were contacted by email (the contact information is public and was collected through the internet): 122 lecturers from the contacted universities decided to participate voluntarily. They followed the instructions of sending a Google Forms questionnaire to their students. Sociodemographic, academic, and lifestyle information was collected to examine the possible effects of sex, age, body mass index (BMI; calculated as weight in kilograms divided by height in meters squared), type of university, academic discipline, academic year, mean marks in the previous year of study, sedentary behavior (minutes/day sitting or reclining), and employment on overall PA and LTPA. Data were collected from April to December 2017.

### 2.3. Measurement of Variables

PA was measured by the Global Physical Activity Questionnaire (GPAQ v2). This questionnaire is a validated questionnaire used to assess PA patterns. The 16 questions on the GPAQ v2 capture information about PA in a typical week [23,24]. Like other self-reporting PA tools, it has shown good test–retest reliability and poor–fair validity [25].

This questionnaire provides information about the total weekly quantity (in minutes) of moderate-to-vigorous PA across three separate domains in which PA is performed: (1) PA in the course of a paid or unpaid job, studies, housework, or a job search (occupational PA (OPA)); (2) PA in the course of getting to work (commuting PA (CPA)); and (3) leisure time PA (LTPA). There is also a question assessing the time (in minutes) spent sitting or reclining during a typical day. We used the Spanish version of GPAQv2, without any content or textual changes.

Overall PA was classified into three levels (high, moderate, and low) based on time spent on PA in a typical week, the number of days, and the intensity of the PA. The GPAQ analysis guide criteria were followed [26] (Table 1). The thresholds for the three levels were based on the recommended PA levels [27]. The criteria for the LTPA level (high, moderate, and low) were the same as for overall PA.

Participants with a low level of PA were deemed inactive. Participants who reported a high or moderate level of PA, i.e., those who achieved the recommended minimum level of PA, were deemed active.

The independent variables were sex, age, BMI, type of university, academic discipline, year of study, mean marks during the previous year of study, minutes spent sitting or reclining, and employment. BMI was categorized according to WHO definitions [28]: underweight (<18.5), normal-weight (18.5–24.9), overweight (25.0–29.9), and obese (>30.0). Universities were classified as public or private. Academic disciplines were stratified using criteria from a report by Spanish universities: social and juridical sciences, engineering and architecture, arts and humanities, health sciences, and physical and life sciences [29]. Mean marks in the previous year of study were divided into four categories: failure (0–4.99), satisfactory (5.00–6.99), good (7.00–8.99), and excellent (9.00–10.00).

### 2.4. Statistical Methods

Questionnaire data were analyzed using the Statistical Package for the Social Sciences (SPSS v21, IBM, Armonk, NY, USA). Descriptive statistics (percentiles, frequencies, and percentages) were calculated to explore the sample’s characteristics. PA levels were calculated using GPAQ v2 procedures. Two binary logistic regressions were performed to analyze the contribution of the independent variables to variance in overall PA and LTPA. Three models were calculated for each dependent variable: (1) nonadjusted; (2) adjusted by sex, BMI, minutes of sedentary behavior, and employment; and (3) fully adjusted. The models’ predictive power was assessed with Nagelkerke’s *r*^2^. The influences of independent variables were expressed as odds ratios. The significance level was set at 0.05.

## 3. Results

### 3.1. Characteristics of the Sample

Table 2 shows the characteristics and distribution of the sample. Median age was 21 years, with an interquartile range of 3 years. The sample was 67.1% female and 32.9% male, and 26.5% had a BMI outside the normal range: 10% were underweight and 16.5% were overweight or obese. Based on overall PA, 77.6% achieved recommended PA levels, but this figure fell to 44.4% when only LTPA was taken into account.

Table 3 presents the percentile distributions of PA data (minutes per week) segmented by domain and sex. Men performed more PA than women, whether overall PA or PA in a specific life domain (OPA and LTPA) was considered.

### 3.2. Associations between Analyzed Variables and the Likelihood of Achieving Recommended PA Levels

Logistic regressions of the likelihood of being active (i.e., achieving recommended PA levels) based on overall PA are presented in Table 4. The likelihood of achieving recommended PA levels was increased by male sex, having a BMI indicative of normal-weight or overweight, work, studying at a public university, and studying health science. These factors accounted for 4.8% of the variance in active status (χ^2^ (15) = 95.45; *p* < 0.001; *r*^2^ = 0.048).

Logistic regressions of the likelihood of achieving recommended PA levels based on LTPA are presented in Table 5. The likelihood of achieving recommended PA levels was increased by male sex; having a BMI indicative of normal-weight or overweight; spending less time sitting or reclining; work; and studying health science, social sciences, engineering, or architecture. These factors accounted for 7.5% of variance in active status (χ^2^ (15) = 171.07; *p* < 0.001; *r*^2^ = 0.075).

## 4. Discussion

This descriptive and cross-sectional study aimed to describe PA levels and the achievement of recommended PA levels based on assessments of overall PA and LTPA in a wide sample of undergraduate students from Madrid and to examine the possible effects of sociodemographic, academic, and lifestyle factors.

The results showed that 22.4% of undergraduate students were not achieving recommended PA levels, based on their overall PA. The differences between studies in terms of instruments used to assess PA, sample characteristics, and statistical treatment made it difficult to compare these results to previous research in this area. Our results differed from those obtained by Cocca et al. [10] in a sample of undergraduate students from Granada (Southern Spain), of whom 60% did not meet recommended PA levels. Varela-Mato et al. [15] also reported a higher prevalence of physical inactivity among undergraduate students in Vigo (Northern Spain), where 72.6% did not meet recommended PA levels. However, there were critical methodological differences between these studies and ours. Unlike us, they did not use the WHO [7] criteria for recommended PA levels shown in Table 1. Cocca et al. [10] classified participants only with time criteria. Participants who performed ≥60 min of PA per day were sufficiently active [30]. Varela-Mato et al. [15] only used an energy expenditure criterion, 600 to 1500 MET min/week.

If, however, we compare our results to those of other studies based on WHO criteria, we find similarities. Research carried out by Calestine et al. [19] with U.S. undergraduate students found that 24% were not achieving recommended PA levels, a similar percentage to our sample. A study of undergraduate students from 23 different countries [14] found that rates of physical inactivity were highest in the Caribbean and South America (37.2%), Sub-Saharan Africa (37.1%), the Near East and Central Asia (32.4%), South Asia and China (45.8%), and Southeast Asia (50.5%). This could be because these universities belong to low- and middle-income countries. There is some evidence that the lower prevalence of physical inactivity in students from high-income countries is due to accessibility to sports, a greater provision of information about healthy habits, and the availability of more and better facilities [14,31]. Our overall PA results are in line with the reported prevalence of physical inactivity in other high-income countries in studies that have used the WHO criteria to define adequate PA. The proportion of students deemed physically inactive was markedly lower in our sample than in university student samples from middle- and low-income countries [14]. However, these data are alarming. The quantity and quality of healthy habits information is growing, but physical inactivity is still high among undergraduate students. Physical inactivity is related to general health problems [2] and worse mental health [32] and is positively related to many factors contributing to the quality of life [33]. The factors that influence physical inactivity during university are not clear, but the promotion of PA could help students to overcome the academic and social challenges associated with going to university [34].

Compared to studies with a general population sample, a study that assessed PA levels from 122 countries concluded that, worldwide, 31.1% of adults (individuals aged ≥15 years) were inactive: that is, their overall PA was below the WHO-recommended PA level. It is well known, however, that PA tends to decrease with age [10,35,36,37], and if we limit our comparisons to studies with samples with a similar age range to our undergraduate sample, Guthold, Ono, Strong, Chatterji, and Morabia [38] found that 13.2% of men aged 18 to 29 years old and 19.1% of women in the same age range were physically inactive. The data on overall PA suggest, therefore, that the proportion of undergraduates who are physically inactive is lower than the proportion of the general population but higher than the general population in the same age range.

If we consider just LTPA, our results showed that 55.6% of participants were not achieving recommended PA levels. This figure for LTPA is much lower than that reported by Haase et al. [31] in a study of 19,298 undergraduate students from 23 different countries, which found that inactivity was most prevalent in Mediterranean countries (39%). Another study of undergraduates from Poland, Slovakia, and Ukraine [39] found that 28.7% of participants were not achieving recommended PA levels during LTPA. In a study with Mauritian students [40], this figure was 40.8%. The proportion of our sample not achieving recommended PA levels during LTPA was higher than in these other studies of undergraduates. University time requirements [1,41] and the influence of the transition to university on students’ habits [11,42] may be reasons that justify the high prevalence of physical inactivity. Likewise, the use of new technology and social networks [19], as well as other unhealthy habits, may be important barriers to the development of PA habits among university students.

Turning to research on general population samples, Meseguer, Galán, Herruzo, and Rodríguez-Artalejo [43] analyzed the temporal trend in LTPA in a wide sample of Spanish adults. They found that the prevalence of physical inactivity increased from 48.7% in 1995 to 59.8% in 2008. These authors also found that just 21.6% of participants in the same age range as undergraduate students (18–24 years) were not achieving recommended PA levels during LTPA [44]. In summary, if we consider just LTPA, it seems that the prevalence of physical inactivity in our sample of undergraduate students was similar to that reported in studies of adult samples but much higher than in more general samples within the same age range.

We also found associations between some of the sociodemographic, academic, and lifestyle variables analyzed and the likelihood of achieving recommended PA levels.

Male students were more likely to be classified as active than female students (i.e., more likely to be achieving recommended PA levels), which is consistent with results from other undergraduate samples [14,45,46,47]. According to LTPA, Haase et al. [31] also found that males were more active than females among undergraduate students from 23 countries. Skidmore and Carson [48] attributed the sex differences in PA levels to differences in psychosocial factors, specifically self-efficacy, social support, and motivation.

Our results also showed that the participants who were employed were more likely to be achieving recommended PA levels, whether it be overall PA or only LTPA. These findings are in line with those of Choi et al. [49], who suggested that professional opportunities and being under supervision were positively associated with active behavior during leisure time. In contrast, Lederer, Autry, Day, and Oswalt [50] suggested that employment has a negative effect on sleep and PA levels.

Turning to lifestyle variables, our results showed that normal-weight and overweight participants were more likely to be achieving recommended PA levels than underweight and obese participants based on overall PA and just LTPA. These results partially corroborated those of Meseguer et al. [44] and Loyen et al. [51], who reported that normal-weight participants, but not overweight participants, were more likely to be achieving recommended PA levels. Even though life habits changes, new schedules, and study requirements contribute to a significant BMI increase [52,53,54,55], it is possible that BMI is not the best predictor of PA for young adults because the components of corporal composition changes are unknown: during this phase of life, the body is changing rapidly [56]. In addition, it could even hide results compatible with physically active behavior (i.e., strength training). On the other hand, our results highlight the negative relationship between sedentary behavior and the achievement of recommended PA levels. This is consistent with previous studies showing that time spent on sedentary activities such as using a computer or watching TV [57,58] or using social media [19] is negatively correlated with volumes of moderate and vigorous PA.

Regarding academic variables, our results showed that undergraduate students of health sciences were more likely to be achieving recommended PA levels than those studying arts and humanities, based on overall PA. Based on LTPA, undergraduate students of social and legal sciences, engineering, architecture, and especially health sciences were more likely to be achieving recommended PA levels than those studying arts and humanities. Perhaps health sciences students are more likely to be achieving recommended PA levels because their studies give them a greater awareness of healthy lifestyle habits. These results were in line with Varela-Mato et al. [15].

Considering overall PA, we found that undergraduates at private universities were less likely to be achieving recommended PA levels than those at public universities. We have not found other published research analyzing the possible relationship between type of university (public or private) and achievement of recommended PA levels in undergraduates, and our results were contrary to what one would expect, since it seems reasonable to assume that students at private universities have a higher income and income is usually positively related to LTPA [59,60].

Our results suggest a need for policies and strategies to promote PA, particularly LTPA, among undergraduate students. With LTPA, we found the greatest discrepancy between observed and recommended levels of PA, and this is also the domain with the greatest scope for action, as noted by Thanamee et al. [61]. The transition to university reduces LTPA [42], and the European Commission has concluded that the three main reasons that stop 15–24-year-old Spaniards from getting sufficient exercise are lack of time (45%), motivation (18%), and high cost (8%) [1]. We suggest that universities should implement programs for PA promotion that make use of university facilities and incorporate PA into the normal academic schedule. Universities should also aim to raise undergraduate awareness of the importance of developing healthy habits, implement programs to facilitate the transition to university, and help students to manage their time better in this new stage of their life. To develop PA among undergraduate students, new ways to promote PA must be considered. For instance, the practice of PA on campus for university credit should be recognized, and the practice of PA and sports for university students or seminars on the promotion of healthy lifestyle habits should be developed. These efforts should be focused on groups that are least likely to meet recommended levels of LTPA: women, students studying subjects outside the health sciences, students who are not employed, and students who are underweight.

Our study had some limitations. The cross-sectional design means that we were unable to infer causal relationships among the analyzed variables. Longitudinal studies would be required to establish cause–effect relationships and track changes in PA levels during students’ university careers. Assessing PA with self-reporting tools is cheap and easy, and the GPAQ has been validated in different countries [25], but it often results in overestimation [62,63], and there are objective methods of assessing PA, e.g., using pedometers and accelerometers that may provide more accurate data. Nevertheless, a methodological strength of this study was the use of the GPAQ as a measurement instrument in a large sample of undergraduates studying in Madrid who were representative in terms of academic discipline.

## 5. Conclusions

Male and female undergraduate students from Madrid who participated in this study were not achieving recommended PA levels, and their levels of LPTA were also insufficient. Sex, BMI, academic discipline, and volume of sedentary behavior were all related to the achievement of recommended PA levels.

## Figures and Tables

**Table 1:** Total physical activity levels and classification criteria recommended for use with the Global Physical Activity Questionnaire (GPAQ). MET: metabolic equivalent of task.

Level of Total Physical Activity	Physical Activity Thresholds
High	≥3 days of vigorous activity (at work and during recreation) in a typical week and a total physical activity MET of ≥1500 min per week
	≥7 days of vigorous activity (commuting, at work, and during recreation) in a typical week and a total physical activity MET of ≥3000 min per week
Moderate	Does not achieve the above criteria but does achieve one of the following:
	(a) ≥3 days of vigorous activity (at work and during recreation) in a typical week, at least 20 min each day
	(b) ≥5 days of vigorous or moderate activity (commuting, at work, and during recreation) in a typical week, at least 30 min each day
	(c) ≥5 days of vigorous or moderate activities (commuting, at work, and during recreation) in a typical week, at least 600 MET minutes per week of total physical activity
Low	Does not achieve the high or moderate criteria

**Table 2:** Sample distribution and characteristics.

Characteristics	*N*	%
Sex		
Female	1987	67.1
Male	973	32.9
BMI		
Underweight	295	10
Normal-weight	2176	73.5
Overweight	385	13
Obese	104	3.5
Employment		
No	2148	72.6
Yes	812	27.4
Type of university		
Public	2363	79.8
Private	597	20.2
Academic discipline		
Arts and Humanities	332	11.2
Physical and Life Sciences	143	4.8
Health Sciences	684	23.1
Social and Legal Sciences	1226	41.4
Engineering and architecture	575	19.4
Year of Study		
1	781	26.4
2	652	22
3	631	21.3
4	700	23.6
5	130	4.4
6	66	2.2
Mean Mark during Previous Year		
Failure (0–4.99)	57	1.9
Satisfactory (5–6.99)	937	31.7
Good (7–8.99)	1728	58.4
Excellent (9–10)	238	8
Achieved WHO-Recommended Level of PA		
No	663	22.4
Yes	2297	77.6
Achieved WHO-Recommended Level Based on LTPA Only		
No	1645	55.6
Yes	1315	44.4

BMI: body mass index; PA: physical activity; LTPA: leisure-time physical activity; WHO: World Health Organization.

**Table 3:** Physical activity descriptive percentiles.

Physical Activity	Percentile						
	5	10	25	50	75	90	95
Total							
Total							
Vigorous	0	0	0	110	270	480	720
Moderate	0	30	120	270	520	920	1350
Work							
Vigorous	0	0	0	0	0	0	180
Moderate	0	0	0	0	0	240	480
Transport	0	0	20	140	280	480	799
Recreational							
Vigorous	0	0	0	90	240	450	600
Moderate	0	0	0	60	180	345	480
Men							
Total							
Vigorous	0	0	7.5	200	400	618	879
Moderate	0	31.2	140	300	564.5	1020	1412
Work							
Vigorous	0	0	0	0	0	0	360
Moderate	0	0	0	0	0	300	557
Transport	0	0	30	150	280	490	750
Recreational							
Vigorous	0	0	0	180	360	540	720
Moderate	0	0	0	60	180	369	540
Women							
Total							
Vigorous	0	0	0	60	225	420	600
Moderate	0	30	120	250	495	900	1332
Work							
Vigorous	0	0	0	0	0	0	96
Moderate	0	0	0	0	0	180	450
Transport	0	0	10	140	280	480	840
Recreational							
Vigorous	0	0	0	30	180	360	492
Moderate	0	0	0	60	180	300	420

Results are expressed in minutes of physical activity per week.

**Table 4:** Logistic regression analyses of the likelihood of achieving recommended PA levels based on overall PA.

Variables	Model 1		Model 2	Model 3
	OR (95% CI)	*r* ^2^	OR (95% CI)	OR (95% CI)
Sex		0.02 ^‡^		
Female	Ref		Ref	Ref
Male	1.85 (1.52–2.26) ^‡^		1.82 (1.49–2.23) ^‡^	1.83 (1.48–2.25) ^‡^
BMI		0.006 ^†^		
Underweight	Ref		Ref	Ref
Normal-Weight	1.56 (1.19–2.04) ^‡^		1.39 (1.06–1.82) *	1.4 (1.06–1.84) *
Overweight	1.76 (1.23–2.5) ^‡^		1.42 (0.99–2.03)	1.46 (1.01–2.1) *
Obese	1.3 (0.78–2.16)		1.07 (0.64–1.8)	1.07 (0.64–1.81)
Sedentary Behavior (min/day)	1 (1–1)	0.001	1 (0.99–1)	1 (0.99–1)
Employment		0.011 ^‡^		
No	Ref		Ref	Ref
Yes	1.6 (1.3–1.97) ^‡^		1.59 (1.29–1.97) ^‡^	1.64 (1.32–2.04) ^‡^
Type of University		0.002		
Public	Ref		Ref	Ref
Private	0.82 (0.66–1.01)		0.81 (0.66–1)	0.74 (0.6–0.93) ^†^
Academic Discipline		0.008 ^†^		
Arts and Humanities	Ref		Ref	Ref
Sciences	1.4 (0.87–2.25)		1.27 (0.79–2.07)	1.33 (0.82–2.16)
Health Sciences	1.64 (1.2–2.24) ^‡^		1.52 (1.1–2.09) *	1.67 (1.2–2.31) ^†^
Social and Legal Sciences	1.1 (0.83–1.45)		0.99 (0.74–1.31)	1.04 (0.78–1.38)
Engineering and architecture	1.28 (0.93–1.75)		1.1 (0.79–1.52)	1.18 (0.85–1.65)
Year of Study	1.03 (0.97–1.1)	0.001	1.02 (0.96–1.09)	1.01 (0.95–1.09)
Mean Marks in Previous Year		0.001		
Failure (0–4.99)	Ref		0 (0–0)	Ref
Satisfactory (5–6.99)	1.18 (0.64–2.18)		1.29 (0.69–2.39)	1.27 (0.68–2.37)
Good (7–8.99)	1.25 (0.69–2.28)		1.45 (0.79–2.67)	1.43 (0.77–2.68)
Excellent (9–10)	1.45 (0.74–2.84)		1.79 (0.91–3.55)	1.86 (0.93–3.71)

Abbreviations: OR: odds ratio; CI: confidence interval; *r*^2^: Nagelkerke’s pseudo *r*-squared value. * *p* < 0.05, ^†^ *p* < 0.01, ^‡^ *p* < 0.001. Model 1: nonadjusted logistic regression. Model 2: logistic regression adjusted for sex, body mass index, volume of sedentary behavior, and employment status. Model 3: fully adjusted.

**Table 5:** Logistic regression analyses of the likelihood of achieving recommended PA levels according to LTPA.

Variables	Model 1		Model 2	Model 3
	OR (95% CI)	r^2^	OR (95% CI)	OR (95% CI)
Sex		0.042 ^‡^		
Female	Ref		Ref	Ref
Male	2.14 (1.83–2.51) ^‡^		2.12 (1.8–2.48) ^‡^	2.1 (1.78–2.48) ^‡^
BMI		0.014 ^‡^		
Underweight	Ref		Ref	Ref
Normal-Weight	1.79 (1.38–2.32) ^‡^		1.56 (1.2–2.02) ^†^	1.58 (1.21–2.05) ^†^
Overweight	2.01 (1.47–2.75) ^‡^		1.58 (1.14–2.19) ^†^	1.63 (1.18–2.26) ^†^
Obese	0.94 (0.58–1.52)		0.79 (0.48–1.3)	0.8 (0.49–1.32)
Sedentary Behavior (min/day)	0.99 (0.99–1) ^‡^	0.007 ^‡^	0.99 (0.99–0.99) ^‡^	0.99 (0.99–0.99) ^†^
Employment		0.002 *		
No	Ref			Ref
Yes	1.19 (1.01–1.4) *		1.17 (0.99–1.38)	1.18 (0.99–1.4)
Type of University		0		
Public	Ref		Ref	Ref
Private	1.09 (0.91–1.29)		1.07 (0.89–1.28)	0.97 (0.8–1.18)
Academic Discipline		0.017 ^‡^		
Arts and Humanities	Ref		Ref	Ref
Physical and Life Sciences	1.54 (1.02–2.31) *		1.32 (0.87–2)	1.28 (0.84–1.95)
Health Sciences	2.32 (1.76–3.06) ^‡^		2.08 (1.57–2.76) ^‡^	2.11 (1.58–2.81) ^‡^
Social and Legal Sciences	1.89 (1.46–2.45) ^‡^		1.71 (1.31–2.22) ^‡^	1.69 (1.29–2.2) ^‡^
Engineering and architecture	1.24 (1–1.53) *		1.4 (1.04–1.89) *	1.4 (1.04–1.89) *
Year of Study	0.98 (0.92–1.03)	0	0.99 (0.93–1.04)	0.96 (0.91–1.02)
Mean Marks in Previous Year		0.002		
Failure (0–4.99)	Ref		Ref	Ref
Satisfactory (5–6.99)	1.26 (0.73–2.17)		1.38 (0.79–2.4)	1.3 (0.74–2.29)
Good (7–8.99)	1.18 (0.69–2.02)		1.4 (0.81–2.43)	1.29 (0.74–2.27)
Excellent (9–10)	0.95 (0.53–1.71)		1.16 (0.64–2.13)	1.12 (0.61–2.07)

Abbreviations: OR: odds ratio; CI: confidence interval; *r*^2^: Nagelkerke’s pseudo *r*-squared value. * *p* < 0.05, ^†^ *p* < 0.01, ^‡^ *p* < 0.001. Model 1: nonadjusted logistic regression. Model 2: logistic regression adjusted for sex, body mass index, sedentary behavior, and employment status. Model 3: fully adjusted.
